# Supplementary material for: Are carbon emissions trading and green financial instruments synergistic? -Comprehensive quantitative research based on content analysis
Source: PLoS One. 2024 Mar 7;19(3):e0298601. doi: 10.1371/journal.pone.0298601 (PMC10919601; doi:10.1371/journal.pone.0298601)
Supplement: S1 Data — (ZIP) [file pone.0298601.s001.zip › Data/地级市Z_F工作报告.zip!/╕ú╜¿/╚¬╓▌╩╨/└┤╫╘╣1⁄2╓┌║┼í╛┬φ┐╦╩2╛▌═°í┐_2014.docx]

各位代表：

　　现在，我代表泉州市人民政府，向大会报告工作，请予审议，并请市政协委员和其他列席的同志提出意见。

　　一、2013年政府工作回顾

　　2013年，在省委、省政府和市委的坚强领导下，我市各级政府深入贯彻落实科学发展观，全力以赴保稳、求进、攻坚，较好地完成市十五届人大二次会议确定的目标任务。

　　——经济运行健康平稳。面对复杂严峻局面，主要经济指标在高基数上实现较快增长。初步统计（下同），全市完成生产总值5250亿元、增长11.5%，公共财政总收入649.6亿元、增长13.5%，公共财政预算收入346.4亿元、增长18%，全社会固定资产投资2500亿元、增长24%，外贸出口158亿美元、增长27%。

　　——软硬实力明显增强。国家“金改区”建设有效推进。当选首届“东亚文化之都”。成功举办2013世界闽南文化节。新对接重点项目342个，总投资3257亿元。一批大项目、好项目建成投产。

　　——城乡建设协调推进。中心城区建成区面积188.5平方公里，城镇化率达61.5%。美丽乡村建设全面铺开。继续荣膺国家卫生城市、全国科技进步先进城市、全国综治“长安杯”等称号。

　　——民生保障有效加强。全市财政民生支出301.8亿元，占公共财政预算支出71.8%。30个为民办实事项目圆满完成。

　　一年来，主要工作和成效是：

　　（一）思忧患谋长远，发展思路更加清晰

　　在市委的直接领导下，深入开展“增强忧患意识 推动长远发展”大讨论活动。组织学习先进城市发展经验，广泛发动社会各界建言献策，高质量完成一批重点调研课题。认真落实市委《关于加快创新转型 推动长远发展的决定》，制定实施加强重大项目工作、推动文化产业发展等一系列政策文件。发展思路进一步提升，政策体系进一步完善，推动发展的正能量进一步汇聚。

　　（二）稳增长调结构，经济运行持续向好

　　应对困难有力有效。发挥政策叠加效应。狠抓各级政策兑现，出台稳工业“8+10”等措施，落实企业帮扶资金19.3亿元、结构性减税和税收奖励35.3亿元，减免行政事业性收费近2亿元。加强经济运行调度。每月召开经济分析会。定期分片区召开企业座谈会，协调解决问题近2000个。组织“万家企业手拉手”等9个专项行动，180家龙头企业与1268家中小企业开展配套协作，对接产能450多亿元。小微企业贷款余额1620亿元，增长24%，同比提高2个百分点。获批农用地转用和土地征收5.3万亩，有效保障项目用地。强化内外市场开拓。成功举办泉州商品博览会、“品牌泉州境外行”系列活动。组织实施鞋服企业去库存。外贸进出口额达280亿美元，口岸通关便利化服务水平评比全省第一。

　　产业升级步伐加快。突出工业支撑。出台推动产业集群发展的意见，安排超过5亿元财政资金支持百个重点转型项目。完成制造业投资912亿元，中化炼油、联合石化“脱瓶颈”、玖龙纸业等52个大项目建成投产或试产。规模以上工业企业利润增长18%，工业对经济增长贡献率达62.3%，亿元工业企业达1880家。深入开展“第三产业发展年”活动。382个项目完成投资551亿元。实现港口货物吞吐量1.1亿吨、集装箱吞吐量170万标箱；泉州晋江机场旅客吞吐量260万人次；新增3A级以上物流企业5家。国家广告产业园区获批建设，入选国家级工业设计中心2家。石狮获评国家级信息消费试点城市。完成旅游总收入447亿元、增长18.8%，获准赴台“个人游”试点，泉台空中直航实现常态化，成功举办茶文化旅游节，仙公山获批国家4A级旅游景区。

　　创新能力不断增强。强化科技创新。培育认定科技小巨人企业311家，新增国家级“两化”深度融合示范企业2家、高新技术企业53家、省级工程技术研究中心16家，引进并启动建设中科院海西研究院装备制造研究所。认真实施“蓝火计划”。“6·18”对接合同项目643个，同比增加108个。新增授权专利1.3万项，制订和修订标准62项。完成高新技术产业增加值650亿元，占地区生产总值比重提高0.9个百分点。强化商业模式创新。获批全国电子商务示范城市。实施电子商务“双推”工程，规划建设电商聚集区7个，设立淘宝网泉州运动品牌馆，全市电子商务交易额超1000亿元、增长60%。菜鸟智能物流东南核心节点项目落户晋江和台商区；中兴外贸电子商务在泉设立总部，跨境电商平台投入试运行；民生电商在泉设立全国纺织、鞋服网络金融事业部。强化人才支撑。列入国家、省引智项目8个，对接海外高级人才项目团队12个，新增博士后工作站4家。出台鼓励大学毕业生自主创业政策措施。

　　（三）抓调度促落实，重大项目强力推进

　　“五大战役”持续深化。完成投资1744亿元，占年度计划的132%。莆永、南石高速建成通车，国道324线晋鲤段拓改、中化青兰山配套码头等竣工投用。泉州湾跨海大桥、海峡石化产品交易市场等扎实推进。国道324线丰洛段拓改、西气东输三线等开工建设。启动城乡基础设施六项“提升工程”。“三维”对接成效明显。深入开展招商大会战。新签约央企项目6个，总投资402亿元。“9·8”投洽会和侨洽会对接项目157个，总投资135.4亿美元，签订5亿美元以上合同项目6个，引进世界500强企业2家。第三届省民企对接会对接项目81个，总投资1343亿元，引进民企500强企业7家。新增泉商回归项目125个，总投资1127亿元。创新项目管理机制。建立重大项目每月一次的协调调度会制度，协调项目96个，绝大多数问题得到及时解决。建立招商项目跨县域落户利益共享机制，实施重大项目招商奖励政策。推行重点项目联合预审和审批代办，提供“月嫂式”服务。出台基本建设项目审批流程改革意见，企业投资项目审批时限压缩到33个工作日。

　　（四）激活力求突破，“两项改革”成效初显

　　“金改区”建设开局良好。认真落实12部委《总体方案》和省政府《实施意见》，出台9份配套文件。实施金改项目67个，完成或部分完成43个。获批消费金融公司试点城市，上报民营银行、泉台合资全牌照证券公司筹建方案。新设银行机构37家、升格2家，设立小微企业金融服务专营机构143家、准金融机构42家，高新区科技金融服务中心和民间借贷登记服务公司投入运营。新增上市企业5家，再融资企业2家，融资总额69亿元。海峡股权泉州交易中心揭牌运营，挂牌企业已达52家。发行债券126.3亿元。建设企业信用信息交换共享平台。成功举办两岸金融合作论坛。金融业实现增加值225亿元、增长18%。“综改区”工作有效推进。认真落实省政府《若干意见》，组织实施10个方面45项重点改革。出台打造九大重点产业链等政策措施。丰泽、晋江服务业和石狮全域城市化、德化统筹城乡发展改革获批省级试点。企业纳税自主申报、按实征收全面推行，“营改增”试点稳步推进。被列为全国城镇低效用地再开发利用试点城市。

　　（五）强基础重统筹，城乡面貌不断改善

　　“三农”工作进一步加强。出台推进现代农业发展、做好扶贫开发的政策措施。全市农林水支出46.6亿元，增长20.7%。加快建设美丽乡村。实施“六大工程”项目3474个，完成投资29.1亿元，实现村庄规划全覆盖，初步建立村庄保洁常态化机制。建设20个市级新农村示范村、20个扶贫开发工作重点村。实施造福工程危房改造3200户。大力发展现代农业。新增省级农民创业园和示范基地9个、设施农业3000亩、“菜篮子”基地11家、林下经济示范点41个和农民专业合作社648家。国家农业科技园获批建设。争取上级渔业补助9亿多元，获批远洋渔业项目2个。设立农业贷款风险补偿资金。完成粮食生产任务。完善农村基础设施。实施水利项目88个，完成投资40亿元。白濑水利枢纽工程前期工作取得进展，彭村水库、晋江防洪工程等16个重大项目扎实推进；治理河道90公里，新解决36.4万名农村人口饮水安全问题。建设升级电力农网1100公里。实施公路安保工程1350公里，改造农村公路265公里和危（病）桥142座。积极应对台风，加强森林防火，防灾减灾成效明显。

　　城市品位不断提升。深化“城市建设管理年”活动，324个项目完成投资351亿元。拓展城市空间。启动环湾县（市）新一轮城市总体规划修编。东海、城东、北峰丰州、晋江滨江、江南、万安、台商区等新区开发加快。实施城市改造更新项目65个，完成投资95亿元。中心市区安置房在建148万平方米，竣工70万平方米。优化景观环境。实施“点线面”攻坚项目85个，完成投资26亿元，建成131公里休闲绿道，初步形成泉州山水慢行系统。建成中心市区供水管网145.6公里。投入1.2亿元开展中心市区防洪排涝整治。持续推进城市内沟河截污。全市改造建设污水管网270公里，新建污水处理厂5座。改进城市管理。率先在全省启用“数字城管”系统平台。深化市政道路和卫生保洁精细化管理，城市卫生综合考评和井盖防坠网加装工程被列为全省典型。交通组织管理进一步优化。出台城市既有住宅增设电梯的指导意见，简化公共维修基金提取程序。深化违法建房专项治理。加快城镇发展。县城和南、北翼新城规划建设扎实推进。21个省市小城镇改革试点镇实施项目730个，完成投资412.5亿元。陈埭、水头两个全国经济发达镇行政体制改革试点顺利实施。

　　生态建设继续强化。成功获批省级生态市。启动重点用能企业能效对标行动，实施节能重点项目141个、减排治理项目1177个，淘汰落后生产线74条，较好完成节能减排任务。出台落实印染行业转型升级意见，加快推进集控区建设和企业整合。实施重点流域水环境整治项目125个，完成投资17亿元；启动晋江、石狮、南安跨境流域污染治理和山美水库流域环境综合整治专项工作；开展21条小流域“赛水质”活动。在全省率先实行最严格水资源管理制度，通过全国节水型社会试点验收。开展“两江”下游非法采砂堆砂专项整治。完成植树造林31万亩、水土流失治理31.6万亩、矿山生态恢复治理74.7万平方米。

　　（六）创特色集亮点，文化发展成绩斐然

　　精心做好首届中日韩“东亚文化之都”竞选工作，成功荣获这一世界级城市名片。精心举办海峡论坛泉州系列活动、第二届世界泉州同乡恳亲大会。完成闽南文化生态保护实验区“十百千基础工程”，建成世界闽南文化展示中心，成立中国社科院闽南文化研究基地。闽南侨批入选世界记忆遗产名录。新增国家级文物保护单位11处、“中国历史文化名街”1条、“中国传统村落”6个。木偶剧获“文华奖”，南音、高甲、歌舞新剧目获国家级大奖，梨园剧团入选全国地方戏创作演出重点院团。城乡公共文化服务体系不断完善，木偶剧院竣工，国家一级图书馆增到7个，基层公共文化设施实现全覆盖。泉籍运动员在体操世锦赛和全运会上取得优异成绩。文博会签约项目18个、合同金额30亿元。新增文化产业示范基地21家，文化产业增加值269亿元、增长20%。

　　（七）惠民生增和谐，社会建设同步加强

　　不断健全社保体系。新增城镇就业15.1万人，农村劳动力转移就业6.1万人。城镇职工“五险”参加人数累计增加36.4万人次。城镇居民医保和新农合参保率分别达95.5%和99.9%，筹资水平和补偿标准进一步提高，实现厦漳泉城镇职工医保卡三地通用。城乡居民养老保险参保率达98.5%。建设提升乡镇敬老院24所、社区居家养老服务站150个、农村老年活动中心20个，创建农村居家养老服务站24个。新开工保障性安居工程3.5万多套，配租配售8000多套。新增归集住房公积金43.8亿元，发放贷款33.3亿元。新增平价商店34家，物价保持稳定。全市慈善组织到位善款5.9亿元，侨捐公益事业5亿元。优先发展教育事业。新改扩建公办幼儿园79所。7个区（市）通过国家义务教育基本均衡发展验收，义务教育标准化学校建设完成率达92%。城区中小学新增学位1.8万多个。五中城东新校区正式招生。福建农林大学安溪茶学院建成，福州大学石油化工学院正式招生，泉州工程职业技术学院获批设立。在全国率先实现外省籍学生在流入地参加高考。加快提升卫生事业。出台推进社会资本举办医疗机构的实施意见。全市乡镇卫生院、社区卫生服务中心和行政村卫生所实现药品销售零差率全覆盖。石狮公立医院改革试点经验在全国推广。落实方便群众就医措施。市第一医院城东院区全部投用，二院东海新院基本建成，全市新增医院床位2350多张。加强计生优质服务和“三高”综合整治，人口自然增长率7.9％。。全力保持和谐稳定。深化“平安泉州”建设，建立县乡村三级巡逻防控体系。深入实施“六五”普法。构建“大调解”工作体系。完善领导干部接访下访制度，认真落实信访“路线图”，信访总量下降9.3%。累计发放流动人口居住证124万张。创建国家安全发展示范城市，开展安全生产大检查、大整治，四项指数全面下降。食品安全专项整治成效明显。精神文明创建取得新成绩。扶持驻泉部队“两中心一基地”、“四个一好”工程建设，驻泉部队官兵积极参与驻地抢险救灾、“平安泉州”和“美丽乡村”建设，省级双拥模范城命名表彰数量位居全省首位。民族宗教、防震减灾、国防动员、民兵预备役、海防、人防、支前、统计、审计、方志、气象、老龄、老干部、老区、残疾人、关心下一代等工作都取得新成绩。

　　（八）优服务提效能，依法行政深入推进

　　认真执行市人大及其常委会决定决议，自觉接受法律监督和工作监督，积极配合市人大常委会开展执法检查、人大代表视察及专项调研；把办理代表建议意见作为“法定作业”，切实回应代表关切，办理人大代表建议336件，满意和基本满意率相加达100%。支持市政协履行职能，主动接受政协民主监督；办理政协委员提案576件，满意和基本满意率达100%。开展国家级行政服务标准化试点，窗口即办率提高至60.4%。建立完善市级政府投融资体系，规范资金统筹和管理。设立市公共资源交易中心。启动市级公务卡结算制度改革。认真贯彻“八项规定”，着力整治“四风”，加强廉政风险防控、行政监察、机关效能、绩效考评等工作。

　　各位代表！成绩来之不易，这是省委、省政府正确领导、关心支持的结果，是市委带领全市人民团结一致、奋力拼搏的结果，是市人大、市政协和社会各界支持监督、共同努力的结果。在此，我代表市人民政府，向全市广大干部群众，人大代表、政协委员，各民主党派、工商联、无党派人士、各人民团体、离退休老同志，驻泉省部属单位、驻泉部队、武警官兵，企业界人士和来泉创业务工人员，以及广大港澳同胞、台湾同胞、海外侨胞、海内外朋友，表示衷心的感谢和崇高的敬意！

　　我们也清醒地认识到，我市发展中还存在不少困难和问题。主要是：经济下行压力加大，工业增加值、社会消费品零售额、城镇居民人均可支配收入未能完成年度预期目标。转型升级任重道远，高新技术产业、现代服务业、新型业态规模偏小。重大项目支撑不足，好项目、大项目不多，融资、征迁、审批等保障不够到位，“落地难”问题还比较突出。企业经营困难增多，商业模式创新不快，市场开拓压力加大。城乡规划建设和管理水平有待提升，中心城区辐射不强、配套滞后，农业农村基础仍较薄弱。环境保护和生态建设任务依然繁重。民生事业人均保障水平不高。社会治理面临一系列新问题新挑战。政务环境仍需改进，少数公务人员担责意识和服务能力不强。对此，我们要采取更加有力的措施加以解决。

　　二、扎实做好2014年政府工作

　　2014年是实施“十二五”规划的关键一年。政府工作的总体要求是：高举中国特色社会主义伟大旗帜，以邓小平理论、“三个代表”重要思想、科学发展观为指导，全面贯彻落实党的十八大、十八届三中全会和习近平总书记系列重要讲话精神，坚持稳中求进、改革创新，加快经济转型、社会转型、城市转型、政府转变，实施“深化五大战役重点行动计划”，全力打造泉州经济升级版、改革创新示范区、生态宜居幸福城。

　　经济社会发展的主要预期目标是：地区生产总值增长10.5%左右，农业总产值持平，工业增加值增长12%；公共财政总收入增长12%，公共财政预算收入增长12%；全社会固定资产投资增长20%；外贸出口增长10%，实际利用外资（验资口径）持平；城镇登记失业率控制在2%以内；社会消费品零售总额增长14%，居民消费价格涨幅控制在3.5%左右；人口自然增长率控制在省下达指标之内；城镇居民人均可支配收入增长9%，农民人均纯收入增长9.5%，完成年度节能减排任务。

　　实现上述目标，我们必须坚持解放思想求先行，创造性地贯彻落实上级决策部署；必须坚持大胆改革激活力，大力破除各种体制机制障碍；必须坚持创新转型促发展，推动经济提质增效；必须坚持民生优先创和谐，让发展成果惠及全市人民；必须坚持海纳百川增合力，充分调动广大干部群众建设现代化泉州的积极性和创造性。重点抓好以下八个方面工作：

　　（一）突出创新转型，打造经济升级版

　　拓展提升先进制造业。实施“产业转型升级行动计划”，加快培育纺织服装、鞋业、石化3个超2000亿元，机械装备、建筑建材、食品饮料3个超1000亿元，工艺制品、纸业印刷、光电信息3个超500亿元产业集群。坚持抓龙头、优增量，大力推进联合石化环氧乙烷及乙二醇、恒安智能化生产基地、荔丝化纤、百宏化纤、辉锐石墨烯制品等项目建设。实施“存量提升工程”，推动产品、工艺、品牌和管理创新；落实 “五年千项”技改行动，年度技改投资610亿元以上。深化“万家企业手拉手”行动。加快“两化”深度融合，组织实施“数控一代”及自动化技术创新专项。推动文化创意、工业设计与制造业的有机结合。尽快推出石材价格和景气指数。建好各类国家级质检中心，争创全国质量强市示范城市。

　　做大做强现代服务业。深化“第三产业发展年”活动，培育壮大生产性服务业。物流业要重点抓好菜鸟智能物流、普洛斯物流园区等项目，应用信息化、网络化提升传统物流业。有效引导外挂车船回迁。商贸业要重点抓好各类全国性、区域性大型专业市场的规划建设和转型升级。旅游业要重点抓好清源山5A级景区、惠安崇武半岛、台商投资区蓝色海湾、德化九仙山旅游度假区等项目，建设三大旅游精品，推进景区“串珠成链”。打造工业旅游品牌。信息服务业要重点抓好EC国际数据中心、泉州软件园、石狮纺织服装创新发展云等项目。

　　培育壮大海洋经济。积极发展海工基地、临港工业、港口物流、海洋生物医药。抓好华宝和万弘海洋生物科技、泉州船厂、嘉信游艇等34个项目。扶持发展海洋运输，支持开辟更多国际国内航线。推进海洋资源市场化配置。加快渔港建设提升，壮大远洋捕捞，发展现代渔业。

　　大力推进科技进步。建好国家高新区，壮大科技小巨人群体，加快发展战略性新兴产业。抓好中科院海西研究院泉州装备制造研究所、“泉州科技云”等创新平台建设。启动制造业关键共性技术“燎原计划”，成立市产学研促进会，建设海峡技术转移泉州中心。抓好国家知识产权示范城市工作。深入实施“海纳百川”高端人才聚集计划，发挥博士后工作站、院士专家工作站、留学归国创业人员联谊会等载体作用，培养引进更多高端人才。扶持发展人力资源服务业。进一步支持大学生自主创业和就业。

　　加快商业模式创新。鼓励企业大力发展电子商务，培育电商品牌、大众品牌。组织特色产品设立网上品牌专区，推动有条件的电子商务园区争创全国示范基地。引导企业立足细分行业，发展专业化的全国性电商平台。抓好中兴外贸电商、闽港跨境电商、两岸贸易电商等项目。推动制造业企业上下延伸、左右拓展，实现从卖产品到卖设计、卖服务等领域延伸，拓展增值空间。

　　提高对外开放水平。发挥2个国家级外贸转型升级示范基地和4个省级出口基地的作用，扩大“三自两高”产品出口，建设出口产品质量安全示范区。继续办好泉州商品博览会和“品牌泉州境外行”系列活动。用好外贸企业融资风险共担资金池、跨境贸易人民币结算、出口信保等政策，健全外贸风险预警体系。支持企业设立境外营销网点，开展国际商标注册和国际品牌并购，培育本土跨国企业。扩大先进技术、关键设备和零部件进口。推广关检合作“三个一”通关新模式，促进一批重点企业货源回归、就地通关。推动石井港区口岸对外开放。做好友城工作。积极参与丝绸之路经济带、21世纪“海上丝绸之路”建设，构建千年古港开放型经济新优势。

　　密切泉台港澳侨合作。紧密对接海峡两岸服务贸易协议，深化泉台经贸文化交流合作。大力推进台商区产业发展、城市建设、对台招商，力争在引进重大台资项目上取得新突破。支持发展对台空中、海上直航。认真做好“内地与港澳更紧密经贸关系安排示范城市”工作，借助港澳平台，扩大开放领域。充分挖掘侨力资源，关心侨亲事业，凝聚侨心，汇聚侨智，维护侨益。

　　（二）突出先行先试，打造改革创新示范区

　　深化金融改革。以服务实体经济作为改革的本质要求，坚持项目化运作，认真落实国家金改新政。引进银行业金融机构2家以上，实现村镇银行县域全覆盖。加快消费金融公司、泉台合资全牌照证券公司筹建，争取设立民营银行、金融租赁公司试点。加强金融产品创新，大力发展小微金融和互联网金融。建成用好金融信息综合服务平台。新增上市企业8家、发债企业8家以上，海峡股权泉州交易中心挂牌企业突破300家，支持企业进入“新三板”挂牌融资。积极稳妥发展准金融机构。建立民间融资网络登记备案制度，促进民间融资阳光化，防范金融风险。

　　拓展民综改革。出台加快民营经济综合配套改革等政策措施，积极争取获批国家发改委试点，打造民营经济发展乐园。支持民间资本进入基础产业、基础设施、社会事业领域。鼓励民营企业进行股份制改造，建立现代企业制度，开展兼并重组。启动千名企业家和百名管理者素质提升培训三年计划，引导做好代际传承工作。抓好工商登记制度改革省级试点，实行“先照后证”、注册资本认缴登记制等措施；进一步做好“个转企”工作，推动符合基本条件的无照企业开展工商登记，壮大经济群体。支持和指导各县（市、区）开展各具特色的主题改革。

　　推进新型城镇化。抓好与全省新型城镇化规划的对接，出台促进城镇化健康发展政策措施。设立环湾城市规划委员会和同城化专门协调机构，强化环湾区域规划、建设、管理、项目等方面的统筹。加快推进石狮、德化城镇化省级改革试点，抓好晋江新型城镇化等省级试点申报。深化小城镇和经济发达镇改革发展。加快实施石结构房屋改造五年计划，争取获得国家棚户区改造以及省政府一系列政策支持。完善流动人口居住证制度，有序推动农业转移人口市民化，加快推进基本公共服务均等化。

　　抓好其他改革。加快建立全口径的预算体系，加强政府性债务管理和风险防控。推进国有企业深化改革和做大做强，引入各类资本参与改制重组，完善国资监管体制。深化农村综合改革，加快农村集体土地确权登记发证工作，积极稳妥推动土地承包经营权流转，用好城镇低效用地再开发和城乡建设用地增减挂钩政策，推进建立城乡统一的建设用地市场。加快价格改革，进一步完善分类电价和阶梯式、差别化水价制度；抓好行政事业性收费改革，全面实施“阳光价费”工程。

　　（三）突出项目带动，增强长远发展能力

　　持续大招商招大商。加强招商力量，提升招商专业化水平。深化“三维”对接、“回归工程”，全力引进大型央企、知名民企、台湾百大、重点侨商和世界500强企业，主攻高端制造业、行业龙头、产业链重要环节和新型业态项目。建好用好4个国家级、10个省级开发区，加快泉州开发区官桥园区设施配套和招商工作。落实重大项目招商奖励和跨县域落户利益共享政策。坚持以落地检验成效，推广和谐征迁工作法，完善重大项目协调调度、跟踪服务、督查考评制度。谋划征集100项事关长远发展的战略性大项目，分期分批组织实施。

　　强化重点项目建设。安排重点项目486个，总投资8732亿元，其中在建项目320个，年度计划投资840亿元，确保建成100个、开工90个。坚持生产性、生财性项目优先，组织实施制造业和服务业重点项目193个，年度计划投资511.7亿元。实施“城乡基础设施提升行动计划”，安排项目312个，总投资2977亿元，为产业发展、民生改善提供强有力的支撑和保障。

　　完善立体交通网络。确保沈海复线泉州段、机场连接线建成通车，加快泉州湾跨海大桥、泉三高速安溪连接线等建设，力争厦沙高速泉州段上半年开工。加快湄洲湾南岸铁路支线、福厦铁路惠安站及配套工程等建设，抓紧吉永泉铁路泉州段前期工作。抓好肖厝5-6＃、石湖5-6＃、斗尾7＃、秀涂16#码头泊位和泉州湾深水航道二期等项目建设。做好泉州晋江机场改扩建扫尾。

　　（四）突出“三农”工作，推进城乡统筹发展

　　构建新型农业经营体系。推进标准农田建设，推广良种良法125万亩，稳定粮食生产。实施“现代农业加快发展行动计划”，市级财政今明两年安排1.3亿元资金扶持现代农业。建设国家级和省级农业科技园区、农民创业园、示范基地12个，扩大设施农业规模。新增专业大户、家庭农场、合作社、农业企业等新型农业经营主体500个，培育新型职业农民1000人。加强农产品质量安全体系、专业市场和冷链物流设施建设。深化泉台农业合作，加快台湾农民创业园、台湾农业技术交流推广中心建设，办好海峡两岸“农订会”。

　　不断提高农民生活水平。认真落实强农惠农富农政策。大力推进扶贫开发，每年统筹安排1亿元资金，扶持30个市级扶贫开发重点乡镇、300个市级重点村发展，实现年度脱贫5万人以上。全力支持安南永德争取中央苏区县政策。推动“老少边岛”地区加快发展。突出抓好新生代外来务工人员的就业帮扶，加强失地失海农民的就业技能培训，新增农村劳动力转移就业6万人以上。继续实施造福工程。盘活农村土地、林地、海域等资源，壮大村集体经济。

　　加强农村基础设施建设。认真落实美丽乡村建设五年行动计划，抓好5条市级示范线、5个示范乡镇和20个示范村，支持永春创建全省美丽乡村示范县。深入开展家园清洁行动，强化农村环境卫生长效管理。实施90个水利项目，推进晋江防洪、“七库连通”工程、山美水库湖泊保护试点等项目建设及白濑水利枢纽工程前期工作，抓好农田水利建设、中小河流治理、水库和海堤除险加固、水电站扩容增效及防灾减灾等工作。

　　（五）突出产城融合，打造宜居幸福城

　　开展“环湾规划建设年”活动。安排项目440个，年度投资441亿元。精心做好环湾区域城乡一体化、综合交通、城市建筑风貌等规划，推动中心城区单元控规全覆盖。全面推进环湾十大片区开发，抓好虫寻埔-金崎新区改造、潘山启动区、百崎湖片区整治、世贸中心等项目及一批安置房建设。稳步推进 “三旧”改造。出台中心市区安征迁补偿统一规范意见。鼓励建筑企业总部回归，支持建筑业发展壮大。

　　科学布局城市配套。完善中心城市内环、二环及环城高速等道路骨干网络。全力推进国道324线丰洛段改造、普贤路拓改、站前东西大道和站前大道鲤城段等建设。抓紧金屿大桥、北峰至城东通道、刺桐路北拓以及城市轨道交通、有轨电车等前期工作。加快城市公交线路加密、延伸和优化，科学规划建设机动车道、自行车道、步行道和停车场设施，着力缓解“行车难”、“停车难”问题。抓好一批优质学校、综合医院、运动场馆。加强地下空间综合利用开发。实施新一轮内沟河截污及护栏安全加固改造。深化“点线面”攻坚，启动“三边三节点”景观塑造，加快城市绿道山线、泉州植物园等建设，开工观音山公园、滨海公园二期等项目。实施晋江、洛阳江两岸景观改造，打造泉州“外滩”。

　　积极促进产城联动。研究推进“都市型产业发展行动计划”。合理规划商务区、会展区、金融区，加快建设东海、鲤城、晋江等总部经济区及泉州会展中心，壮大总部经济、楼宇经济。积极引进国内外知名开发商参与开发，推动房地产业持续健康发展，建好一批城市和商业综合体项目，促进文化、旅游、健身、家政等服务消费，培育核心商圈。加快推进南、北翼新城规划建设，抓好泉港产城联动发展新城区、南安沿海“三镇”产城联动发展新城镇试点。

　　提升城市管理水平。深化精神文明创建工作，突出抓好公共环境、公共秩序、市民素质提升等关键环节，力争进入全国文明城市行列。加快建设“智慧泉州”，扩大“数字城管”覆盖面。进一步落实市、区、街道管理职责，完善城市管理长效机制，抓好城乡结合部、老旧社区、背街小巷、集贸市场等薄弱区域环境整治，巩固扩大创卫成果。深化城市道路精细化管理，落实临时占用挖掘道路审批公示制度。组织开展打击“两违”专项行动，坚决遏制“两违”。强化城市管理综合考评，进一步发挥市容市貌观察团作用。完善物业管理制度，规范新型社区服务。

　　（六）突出文化引领，建好“东亚文化之都”

　　打响文化品牌。实施古城文化复兴计划，全力办好“东亚文化之都”系列活动，打造影响东亚、面向世界的“多元文化都市”。改造提升泉州影剧院等演出场所，筹建泉州当代艺术馆、歌舞剧院、市图书馆新馆、工人文化宫等项目。抓好闽南文化生态保护区20个重点区域的整体性保护建设。积极推动“海丝”申报世遗工作。大力弘扬泉商文化。

　　壮大文化产业。坚持以文兴业、以文塑城，做大做强十大文化产业。做好历史街巷的保护性开发，推进西街保护整治。实施文化与旅游融合发展示范工程，抓好17个示范项目，推动文化融入旅游、旅游牵手文化。抓好国家广告产业园区、惠安雕艺文创园等建设，培育一批文化产业聚集区。促进文化与科技、金融、商贸深度融合，培育新型文化业态。

　　落实文化惠民。深入开展社会主义核心价值体系教育。深化文化体制改革，统筹发展城乡文化事业。围绕创建国家公共文化服务体系示范区，推进县、乡、村三级文化设施提档升级，创新文化服务模式，推动文化惠民项目与群众文化需求有效对接。加快第二轮修志工作。

　　（七）突出环保优先，创建国家生态城市

　　大力推进节能减排。实施100个重点节能项目，组织134家重点用能企业开展节能低碳行动，坚决淘汰落后产能。强化工业园区污染集中处理，突出重点区域、重点行业、重点企业污染减排治理，抓好污水处理厂及其配套管网建设和运营。加快建设石狮、晋江印染行业循环经济园区，促进印染企业重组整合和转型升级。抓好德化陶瓷国家循环化改造示范试点园区、海西再生资源产业园区及安溪LED照明产品推广应用示范县建设。

　　持续深化环境整治。加强饮用水源保护建设，继续推进中心市区供水管网改造，进一步解决好农村人口饮水安全问题。启动新一轮重点流域专项整治，深化石狮、晋江、南安跨境流域污染治理，抓好洛阳江综合整治开发规划，深化小流域“赛水质”活动。强化机动车尾气、施工扬尘、油烟、工业废气综合治理。加快工业固体废物处置等环保设施建设，加强化学品、危险废弃物处置和监管，防治重金属污染。推进新一轮农村环境综合整治。坚守环保底线，严厉打击各类环境违法行为。

　　创新生态建设举措。实施“宜居环境建设行动计划”，启动省级“森林城市”创建，确保通过国家生态市考核验收，打造“美丽泉州”。加快建立生态文明制度体系，启动排污权有偿使用和交易试点，做好污染源自动监控设施第三方运营维护工作，完善生态补偿机制。抓好永春桃溪流域生态经济试验区省级试点申报工作。实施山体、水域、海岸带生态修复工程，强化水土流失、“青山挂白”、工矿废弃地恢复治理，继续推进“四绿”工程，加强森林防护和生态景观建设。

　　（八）突出民生保障，健全公共服务体系

　　认真做好事办实事。实施“民生工程行动计划”，抓好城市卫生设施、防洪排涝、农村公路提级和危（病）桥改造、特困失能老人帮扶、残疾儿童康复救助、农民健康、低保家庭安居房建设、地质灾害危险点居民搬迁、“菜篮子”基地建设等33项为民办实事项目，从薄弱环节入手，集中力量解决一批事关群众切身利益的问题。

　 完善社会保障制度。建立经济发展和扩大就业的联动机制，大力培养高素质劳动者和技能型人才。抓好城镇职工“五险”、城乡居民养老保险、城镇居民医保和新农合等工作。发展养老服务事业和产业，鼓励居家养老，引导社会力量兴办养老服务机构。启动市社会福利中心建设，支持发展慈善、社会救助和残疾人事业，完善社会救助和保障标准与物价上涨挂钩联动机制，鼓励开展志愿服务活动。认真实施妇女儿童发展纲要，保障妇女儿童合法权益。抓好一批以公共租赁住房为主的保障性住房建设。

　　大力推进教育现代化。深化教育领域综合改革。启动第二轮学前教育三年行动计划。力争创建“教育强区”2个，通过义务教育发展基本均衡县（市、区）省级督导评估6个。加快城东、东海、江南学园建设，力争泉州实小洛江校区、晋光小学东海校区秋季正式招生；增加中小学学位1万个，努力完成义务教育标准化学校创建任务。建成泉州教育城域网。加快现代职业教育体系建设。支持办好华侨大学、泉州师院等高校，从体制机制上解决人才培养与需求“两张皮”问题，提升高校服务地方发展的能力。规范发展民办教育，扶持发展特殊教育、老年教育、继续教育和社区教育。做好关心下一代工作。

　　提升卫生服务水平。深化医改工作，扩大公立医院改革试点。加快市中医联合医院、光前医院扩建、晋江和石狮市医院新院等项目以及妇产、儿童、精神卫生等薄弱学科建设，全市新增医院床位2500张以上。强化乡镇、社区医疗卫生服务功能。抓好卫生人才队伍建设。大力发展中医药事业。深入开展爱国卫生运动，提高疾病防控、妇幼保健、公共卫生服务水平。落实人口计生政策，促进人口长期均衡发展，提高人口素质。开展全民健身活动，全力备战省运会。

　　创新社会治理机制。改进社会治理方式，强化系统治理、依法治理、综合治理、源头治理。深化“六五”普法，建设法治泉州。完善社会信用体系，建立守信激励、失信惩戒制度。创新有效化解社会矛盾机制，完善“大调解”机制和信访“路线图”，做好积案化解工作。深化国家安全发展示范城市创建工作，建立隐患排查治理体系，强化重点领域安全专项整治，提升标准化建设水平，加快建设石化安全生产应急救援中心，遏制重特大事故发生。建立最严格的食品药品监管制度，有效治理“餐桌污染”。完善社会治安立体防控体系，加强执法规范化建设，重拳整治社会治安热难点问题，整合各方面资源和力量，推行城乡社区网格化服务管理模式。促进民族团结、宗教和谐。抓好国防动员、民兵预备役、支前、人防等工作，推进军民融合深度发展，争创全国双拥模范城“八连冠”。

　　三、努力建设法治政府和服务型政府

　　各位代表！加快转变政府职能，创新行政管理方式，提高政府有效治理能力，是时代的新要求，是人民的新期待！

　　我们要解放思想、锐意进取。始终牢记忧患、志在长远，进一步转化大讨论活动成果。以爱拼敢赢的锐气、自我革新的胸怀，坚决破除思维定势、体制障碍，在更高层面上推进泉州改革开放。全面正确履行职能，抓好市、县政府机构改革，推进事业单位分类改革，强化公共服务、市场监管、社会管理、环境保护等职责。完善发展成果考核评价体系，更加注重人的发展，更加注重有质量、有效益、可持续的发展。

　　我们要优化环境、提升效能。坚持有所为、有所不为，把该放的权力放彻底，把该管的事项管到位。推动行政审批事权继续向县级、重点乡镇下放；开展部门审批职权向社会中介组织转移试点。全面实施国家级行政服务标准化试点，推进标准化“四级”对接联动和县乡全覆盖，推行“一表式”全程网上审批。倡导雷厉风行、马上就办。严格问责问效，改进绩效评估。开展“阳光减负”专项行动，清理规范涉企涉农收费和各类检查评比。

　　我们要改进作风、务实为民。扎实开展群众路线教育实践活动，严格执行“八项规定”，坚决整治“四风”，着力解决人民群众反映强烈的突出问题。认真落实《党政机关厉行节约反对浪费条例》和《党政机关国内公务接待管理规定》等党纪党规，全面推行公务卡结算制度。始终坚持根基在人民、血脉在人民、力量在人民，大力推进“四下基层”，积极倡导“一线工作法”，多到困难大、矛盾多、条件差的地方去解决实际难题。

　　我们要依法行政、清正廉洁。牢固树立对人大负责的意识，坚决执行市人大及其常委会决定决议，及时向市人大及其常委会报告工作，依法接受法律监督和工作监督。进一步拓宽协商民主渠道，自觉接受人民政协的民主监督。广泛听取民主党派、工商联、无党派人士意见建议，主动接受媒体监督和社会公众监督。强化行政监察和审计监督。积极构建决策科学、执行坚决、监督有力的权力运行体系。进一步加强行政执法、政务公开、行政复议以及规范性文件审查备案工作。全体公务人员要胸怀大局，知行合一，心存敬畏，严守纪律，以自己的实际行动，促进干部清正、政府清廉、政治清明新风的形成。

　　各位代表！新的一年，困难和挑战考验着我们，责任和使命激励着我们！让我们紧密团结在以习近平同志为总书记的党中央周围，在中共泉州市委的领导下，抓住机遇，趁势而上，开拓进取，奋发有为，为创造泉州更加幸福美好的未来而努力奋斗！
